# Harnessing AI in prosthodontics and implant dentistry: An umbrella review of systematic evidence

**DOI:** 10.1111/jopr.70091

**Published:** 2026-01-14

**Authors:** Amal Alfaraj, Álvaro Limones, Shakil Ahmad, Fahad Aljubairah, Salem Albalaw, Mohammad Albesher, Basel Alghamdei, Wei‐Shao Lin

**Affiliations:** ^1^ Department of Prosthodontics King Faisal University College of Dentistry Al Ahsa Saudi Arabia; ^2^ Department of Prosthodontics Indiana University School of Dentistry Indianapolis Indiana USA; ^3^ Department of Conservative Dentistry and Orofacial Prosthodontics Faculty of Dentistry Complutense University of Madrid (UCM) Madrid Spain; ^4^ Directorate of Library Affairs Imam Abdulrahman Bin Faisal University Dammam Saudi Arabia; ^5^ King Faisal University College of Dentistry Al Ahsa Saudi Arabia

**Keywords:** artificial intelligence, clinical translation, evidence synthesis, implant dentistry, machine learning, prosthodontics

## Abstract

**Purpose:**

To synthesize evidence from systematic reviews on artificial intelligence (AI) applications in prosthodontics and implant dentistry, focusing on clinical applications, AI model performance, and quality of evidence.

**Methods:**

A comprehensive search was conducted in PubMed (MEDLINE), Scopus, Web of Science, Embase, and The Cochrane Library databases, identifying systematic reviews published from 2018 to 2025. Inclusion criteria comprised systematic reviews evaluating AI in prosthodontics or implant dentistry, published in English. Narrative reviews and reviews from other dental specialties were excluded. Two reviewers independently performed study selection and data extraction, and the methodological quality of the included systematic reviews was assessed using A Measurement Tool to Assess Systematic Reviews (AMSTAR‐2) tool. This umbrella review was registered in the International Prospective Register of Systematic Reviews (PROSPERO) database (CRD420251067048).

**Results:**

Eleven systematic reviews were included. AI demonstrated substantial capability in prosthodontics for caries and fracture detection (with an accuracy of ∼82%–89%), automated tooth shade matching, and prosthesis design. In implant dentistry, AI algorithms accurately identified implant types on radiographs (∼95.6% pooled accuracy), optimized implant placement planning, and predicted treatment outcomes with moderate accuracy (62.4%–80.5%). Performance was strongest for radiographic identification and anatomic segmentation tasks in implant dentistry. It was more modest for preparation margin detection and objective shade matching in prosthodontics, as well as for multivariable prognosis and for detecting maxillary edentulous sites in implant dentistry. Convolutional neural networks (CNNs) consistently outperformed traditional algorithms in image‐based tasks. However, AI prediction of long‐term outcomes showed moderate performance due to data limitations and biological variability. Overall, although four reviews were rated as high quality, the majority exhibited low or critically low methodological quality, primarily due to a lack of a priori protocol registration and incomplete bias assessment.

**Conclusion:**

AI applications in prosthodontics and implant dentistry may enhance diagnostic and planning workflows, especially for recognition and segmentation tasks. Nevertheless, most evidence comes from early‐stage, retrospective, or highly controlled studies, highlighting the need for prospective clinical validation and higher‐quality systematic reviews before routine clinical adoption can be recommended.

Historically, prosthodontics and implant dentistry have heavily relied on clinicians' expertise and meticulous manual skills.^1^ Advancements in digital technologies, including intraoral scanning, cone‐beam computed tomography (CBCT), computer‐aided design and computer‐aided manufacturing (CAD‐CAM), and 3D printing, have increasingly reshaped prosthodontics and implant dentistry, enabling precise diagnostics, treatment planning, and prosthesis fabrication.^2^ These digital tools have significantly improved clinical workflows, reduced treatment times, and enhanced patient outcomes through improved prosthetic accuracy and patient‐specific customization.^3^

Artificial intelligence (AI) has emerged as a transformative technology in healthcare, leveraging large datasets, including clinical images, radiographs, and patient records, to support diagnostics, treatment planning, decision‐making, and prognostics.^4^ Machine learning (ML), deep learning (DL), convolutional neural networks (CNNs), and artificial neural networks (ANNs) represent key AI methodologies extensively adopted within dentistry. These methods offer powerful pattern recognition capabilities and predictive accuracy, often matching or exceeding human clinicians in specific diagnostic and predictive tasks.^5^ Prosthodontic applications of AI include automated detection of dental caries, identification of vertical root fractures, and tooth preparation margin delineation from digital scans.^6^ Such AI‐driven diagnostics have demonstrated robust accuracy and reliability, potentially reducing clinician subjectivity and diagnostic variability. Additionally, AI technologies are increasingly used in prosthetic shade selection, automated prosthetic design, and optimization of prosthetic fitting, thereby enhancing clinical outcomes, esthetic results, and operational efficiency in dental laboratories.^7^

In implant dentistry, AI has been leveraged primarily in radiographic diagnostics and surgical planning. DL models, particularly CNNs, have consistently demonstrated high accuracy in identifying dental implant types from radiographs, providing valuable diagnostic support in cases where implant documentation is unavailable. Moreover, AI‐based anatomical segmentation of critical structures on CBCT images, such as mandibular canals and maxillary sinuses, has significantly improved surgical planning precision and patient safety. AI‐driven finite element analysis has also optimized implant design parameters, effectively reducing biomechanical stress at implant‐bone interfaces, potentially improving implant longevity and clinical performance.^8^

Despite promising outcomes, AI integration in prosthodontics and implant dentistry remains in developmental stages, although individual studies highlight AI's clinical potential. Numerous systematic reviews have recently emerged, evaluating specific AI applications and performance metrics; however, there is a lack of integrated evidence to inform clinical practice.^9^ An umbrella review, or overview of systematic reviews, offers a higher‐level synthesis approach, integrating multiple systematic reviews into a unified analytical framework. Such an approach is particularly valuable in rapidly evolving fields such as AI, where studies proliferate quickly, evidence becomes fragmented, and clinicians require comprehensive and coherent evidence syntheses to guide clinical decision‐making.^10^ To date, no comprehensive umbrella review has been conducted specifically to evaluate AI applications in both prosthodontics and implant dentistry.

This umbrella review aimed to address the primary research question structured according to the PICO framework: “In patients undergoing prosthodontic or implant dental treatment (P), do the artificial intelligence‐based tools (I), compared with no intervention (absence of AI tools) (C), achieve clinically acceptable performance in key clinical tasks such as diagnostic accuracy, treatment planning, clinical efficiency, and patient‐related clinical outcomes (O)?” To systematically answer this question, the review categorizes clinical AI applications within these dental specialties, summarizes reported AI model performances using standardized metrics, evaluates the methodological quality of included reviews using A Measurement Tool to Assess Systematic Reviews (AMSTAR‐2), and identifies critical knowledge gaps to provide actionable recommendations for clinical implementation and future research directions. Given that direct comparisons with conventional workflows were rarely available, the overarching objective of this umbrella review is to assess the clinical readiness and potential acceptability of current AI applications for these tasks, rather than to claim proven superiority over conventional workflows.

## METHODS

### Protocol and registration

This umbrella review was reported according to the Preferred Reporting Items for Overviews of Reviews (PRIOR) 2022 guidelines.^11^ A detailed protocol was prepared a priori and registered in the International Prospective Register of Systematic Reviews (PROSPERO) under the ID CRD420251067048. The review protocol specified objectives, eligibility criteria, search strategy, data extraction, and quality assessment procedures to evaluate systematic reviews examining AI applications in prosthodontics and implant dentistry.

### Literature search

A comprehensive literature search was conducted in April 2025, following the prospective registration of the review protocol, to identify eligible systematic reviews. Electronic databases, including PubMed, Scopus, Web of Science, Embase, and the Cochrane Database of Systematic Reviews, were systematically searched. The search strategy combined Medical Subject Headings (MeSH) terms and relevant keywords in the domains of AI, prosthodontics, and implant dentistry. The detailed search strategy for each database is provided in Table 1. Terms included “artificial intelligence,” “machine learning,” “deep learning,” “neural networks,” “CNN,” “ANN,” “prosthodontics,” “dental implants,” “CAD/CAM,” and “digital dentistry,” among others. To ensure comprehensive coverage, additional manual searches were performed on the reference lists of included systematic reviews, and citation tracking was conducted through Google Scholar. Searches were restricted to systematic reviews published from January 1, 2018, to April 30, 2025, to reflect recent advances in dental AI. Only English‐language publications in peer‐reviewed journals were considered. Duplicates were managed and removed using reference management software (Mendeley; Elsevier Ltd).

**TABLE 1:** Search strategy.

Database	Search terms	Filters applied
**PubMed**	(“Artificial Intelligence” [MeSH] OR “machine learning” OR “deep learning” OR “neural networks” OR “CNN” OR “ANN”) AND (“prosthodontics” OR “dental prosthesis” OR “dental implants” OR “CAD/CAM” OR “digital dentistry”)	English, 2018–2025
**Scopus**	TITLE‐ABS‐KEY (“artificial intelligence” OR “machine learning” OR “deep learning” OR “convolutional neural network”) AND (“prosthodontics” OR “implant dentistry” OR “digital prosthesis” OR “CAD/CAM”)	English, peer‐reviewed, 2018–2025
**Web of Science**	TS = (“artificial intelligence” OR “machine learning” OR “deep learning” OR “CNN” OR “neural networks”) AND TS = (“prosthodontics” OR “dental implants” OR “digital prosthetics”)	Articles, English, 2018–2025
**Embase**	(“artificial intelligence”/exp OR “machine learning” OR “deep learning”) AND (“prosthodontics”/exp OR “dental implants”/exp OR “digital dentistry”)	English, 2018–2025
**Cochrane Library**	(“artificial intelligence” OR “machine learning” OR “deep learning”) AND (“prosthodontics” OR “implant dentistry” OR “dental prosthesis”)	Systematic Reviews, English, 2018–2025
**Other sources**	Manual search of reference lists and citation tracking via Google Scholar	N/A

### Inclusion and exclusion criteria

Systematic reviews were eligible if they evaluated AI applications specifically within prosthodontics or implant dentistry. Eligible reviews were required to report a database search and predefined eligibility criteria; reviews with incomplete methodological detail were included but rated lower on AMSTAR‑2.^12^ Reviews addressing diagnostic tasks, treatment planning, prosthetic design, outcome prediction, and clinical decision support were included. Included systematic reviews had to focus explicitly on prosthodontic treatments, including fixed, removable, maxillofacial, or digital prosthetics, or dental implant procedures involving implant placement, identification, optimization, or outcome prediction. Only systematic reviews published in English from 2018 to 2025 were included. Exclusion criteria encompassed non‐systematic methodologies such as narrative reviews, editorials, commentaries, conference abstracts, expert opinions, and case reports. Reviews specifically examining orthodontics, periodontics, endodontics, pediatric dentistry, oral radiology, or general dental applications outside prosthodontics or implant dentistry were excluded.

### Study selection and data extraction

Two reviewers (F.A. and S.A.) independently screened titles and abstracts from the initial database searches against predefined inclusion criteria. Articles deemed potentially eligible underwent full‐text assessment independently by both reviewers. Any disagreements regarding study eligibility were resolved through discussion or consultation with a third reviewer (A.A.).

Data extraction was independently performed by two reviewers (F.A. and S.A.) using a standardized extraction form specifically developed for this review. Extracted information included authors, publication year, journal details, clinical domains (prosthodontics, implant dentistry, or combined), number and type of primary studies included AI applications (diagnosis, planning, design, outcome prediction), AI model types (CNN, ANN, SVM, ensemble methods), performance metrics (accuracy, sensitivity, specificity, predictive values), primary outcomes, and reported limitations. Details about the meta‐analytic methods used and the risk of bias assessment for primary studies were also recorded. Information on funding sources of the included systematic reviews was extracted where reported. The extracted data were cross‐validated for accuracy, and discrepancies were resolved by consensus with a third reviewer (A.A.). Overlapping primary studies identified across multiple systematic reviews were considered to minimize duplication bias during synthesis.

### Quality assessment

The methodological quality of each systematic review was critically appraised using the AMSTAR‐2 tool.^12^ Two authors (M.A. and B.A.) of this umbrella review independently assessed each review, with disagreements resolved by discussion with a third reviewer (A.A.). This quality appraisal is summarized in Table 2.^6^, ^8^, ^9^, ^13^, ^14^, ^15^, ^16^, ^17^, ^18^, ^19^, ^20^ Overlap among primary studies cited across the included systematic reviews was quantified using the corrected covered area (CCA) method. First, an overall CCA was calculated across all 11 reviews to describe the extent of primary‐study overlap at the umbrella‐review level. In a second step, task‐level CCA was calculated for each clinical AI task or outcome addressed by at least two data‐providing systematic reviews by mapping the contributing primary studies across the reviews. When only one systematic review contributed data for a given task, or when differences in eligibility criteria, language restrictions, or task stratification prevented reliable matching of primary studies, task‐level CCA was considered not applicable (N/A). Task‐level CCA values and justifications are reported and interpreted solely as descriptive measures of primary‐study overlap, not as indicators of AI performance.

**TABLE 2:** Characteristics of included systematic reviews on AI in prosthodontics and implant dentistry.

Systematic reviews on AI in general prosthodontics (tooth‐supported prostheses and overall prosthodontic workflows)						
No.	First author (Year)	Review focus and scope	Included studies (*n*)^*^	Meta‐analysis	Quality^**^	Notable quality issues
1.	Bernauer et al.^14^	AI applications in prosthodontics (overall use and performance in fixed, removable, implant prosthetics)—broad scope, last 5 years.	7 studies	No (Descriptive)	Critically low	Methods not pre‑registered; study selection not reported in duplicate; no full list of excluded studies; funding of included studies not reported; risk of bias not incorporated into interpretation.
2.	Revilla‐León et al.^6^	AI models for tooth‐supported fixed and removable prosthodontics—tasks: shade selection, restoration design, margin line, casting, facial changes, RPD design.	36 studies	No (Descriptive)	Critically low	Methods not pre‑registered; study selection not reported in duplicate; no full list of excluded studies; funding of included studies not reported.
3.	Maktabi et al.^17^	Role of AI in prosthodontics (effectiveness and success)—review of AI in removable, fixed, maxillofacial, and implant prosthodontics.	9 studies	No (Descriptive)	Low	Research question not framed using PICO; no pre‑registered protocol; non‑comprehensive search; duplicate study selection and data extraction not reported; included studies not described in adequate detail; no excluded‑study list, risk‑of‑bias assessment, or heterogeneity discussion; funding not reported.

Systematic reviews on AI in implant dentistry						
No.	First author (Year)	Review focus and scope	Included studies (*n*)	Meta‐analysis	Quality^*^	Notable quality issues
4.	Revilla‐León et al.^8^	AI applications in implant dentistry—including implant type recognition, implant success prediction, and implant design optimization.	17 studies	No (Descriptive)	Critically low	Methods not pre‑registered; study selection not reported in duplicate; no full list of excluded studies; funding of included studies not reported.
5.	Alqutaibi et al.^9^	AI algorithms for dental implant identification (system type recognition on radiographs)—includes diagnostic accuracy meta‐analysis.	22 studies	Yes (Accuracy)	High	High‑quality methods overall, but included studies were not described in full detail and funding sources for primary studies were not reported.
6.	Alqutaibi et al.^18^	AI in dental implant treatment planning (edentulous area detection and bone measurements on imaging)—includes meta‐analysis.	12 studies	Yes (Accuracy)	High	High‑quality methods overall; minor issues include limited descriptive detail on included studies and lack of funding information for the primary studies.
7.	Alqutaibi et al.^20^	AI algorithms for predicting dental implant prognosis (from radiographic indicators)—diagnostic accuracy for outcomes like implant failure, peri‐implantitis.	13 studies	No (Quantitative synthesis)	High	High‑quality methods overall; included studies were not described in full detail and funding sources for primary studies were not reported.
8.	Bonfanti‐Gris et al.^15^	AI for implant classification and peri‐implant pathology on 2D radiographs—identification of implant brands and detection of peri‐implant diseases.	29 studies	No (Descriptive)	High	High‑quality review; main AMSTAR‑2 limitation is lack of reporting of funding sources for the included primary studies.
9.	Macrì et al.^19^	AI in dental implant planning (systematic review in Bioengineering)—focus on AI for anatomic structure identification (CBCT imaging) and implant positioning.	14 studies	No (Descriptive)	Critically low	No pre‑registered protocol; data extraction not reported in duplicate; no full list of excluded studies; funding sources of included studies not reported.
10.	Dashti et al.^13^	Accuracy of AI (DL vs. conventional models) in dental implant type detection on radiographs—systematic review with meta‐analysis.	13 studies (3 in meta‐analysis)	Yes (Accuracy, Sens/Spec)	Critically low	Despite otherwise rigorous methods, the review lacked a pre‑registered protocol, did not provide a full list of excluded studies, and did not report funding of included studies.
11.	Ibraheem et al.^16^	Accuracy of AI models in dental implant fixture identification and classification (Diagnostics journal review).	21 studies	No (Descriptive)	Critically low	Did not provide a full list of excluded studies and did not report funding sources for the included primary studies.

* “Included studies (*n*)” reflects the number of studies in the main analyses reported by each review, which differs from the total number of primary‑study citations (*N* = 281) used to calculate overall CCA.

** Quality categories (high, moderate, low, critically low) were assigned using the AMSTAR‐2 tool, based on weaknesses in critical and non‐critical methodological domains: high = no or one non‐critical weakness; moderate = more than one non‐critical weakness but no critical flaws; low = one critical flaw; and critically low = more than one critical flaw.

### Data synthesis

Due to substantial heterogeneity in AI applications, clinical tasks, study designs, and outcome measures across the included reviews, statistical pooling of results across reviews (i.e., meta‐analysis of meta‐analyses) was not appropriate. In most cases, the included systematic reviews did not provide sufficiently comparable outcome metrics to support quantitative synthesis at the umbrella‐review level. Accordingly, a descriptive synthesis was undertaken, with the evidence summarized narratively and key findings presented in structured tables, organized either by review article (Table 3)^6^, ^8^, ^9^, ^13^, ^14^, ^15^, ^16^, ^17^, ^18^, ^19^, ^20^ or by clinical task (Table 4).^6^, ^8^, ^13^, ^14^, ^15^, ^16^, ^18^, ^19^, ^20^

**TABLE 3:** Summary of findings from included systematic reviews on AI in prosthodontics and implant dentistry.

Review (Author, Year)	Key findings	Authors' conclusions
1. Bernauer et al.^14^	Very few AI studies in prosthodontics (7 total) met inclusion. Demonstrated applications in CAD/CAM (design of prostheses), implant prosthetics, tooth preservation prognosis, and orofacial analysis. No meta‐analysis; all results descriptive.	AI shows *potential across various prosthodontic tasks*, but usage is still rare. Emphasized need for more clinical studies and noted rapid digital tech turnover, AI tools must be continually validated and updated.
2. Revilla‐León et al.^6^	Reviewed 36 studies in 6 categories: Shade matching (1 study): AI better than visual selection; Restoration design (14 studies): AI‐generated designs feasible for crowns/fixed dental prosthesis Margin line detection (1 study): CNN marked prep margins with ∼90%–97% accuracy Casting optimization (2 studies): AI improved casting quality (less porosity, faster) Facial changes prediction (1 study): AI predicted patient facial profile with dentures RPD design and education (17 studies): AI used for RPD framework design support and student training (expert systems, interactive programs)	*AI can reliably perform many prosthodontic tasks*: it improves objectivity (shade selection), automates labor‐intensive steps (design, margin marking), and can act as a decision‐support or training tool. However, these AI models are largely prototypes—additional studies are needed to confirm their clinical performance. Authors call for integrating AI into dental workflows cautiously, alongside expert oversight.
3. Maktabi et al.^17^	Included 9 studies on AI in removable, fixed, implant prosthodontics. Reported positive performance outcomes when AI was applied, such as improved efficiency in denture fabrication, enhanced accuracy in diagnostics, and patient satisfaction tools (AI for patient communication/anxiety reduction). Noted that “prosthodontic implant applications” benefitted most from AI advances.	*AI is increasingly used in prosthodontics* and enhances rehabilitation outcomes by reducing human error and improving workflow efficiency. The review reinforces that all subfields (removable, fixed, maxillofacial, implant) can gain from AI, with implant dentistry leading the way. They recommend more comprehensive documentation and standardization in future AI research, and suggest educating clinicians about AI tools for better adoption.
4. Revilla‐León et al.^8^	Analyzed 17 studies: Implant type recognition (7 studies): AI (mostly CNNs on radiographs) achieved 93.8%–98% accuracy in identifying implant systems Implant success prediction (7 studies): AI models using clinical/radiographic data predicted implant survival with 62.4%–80.5% accuracy (moderate performance) Implant design optimization (3 studies): AI + FEA improved implant designs (∼36% stress reduction at bone interface with AI‐optimized design).	*AI has great promise in implant dentistry* for identification, risk prediction, and even design innovation. However, these models are still in development, particularly outcome prediction needs improvement (current accuracy ∼70%). Authors stress the need for further studies with larger datasets and clinical validation, as well as combining AI with biomechanical modeling (as shown in design optimization) for next‐generation implants.
5. Alqutaibi et al.^9^	Included 22 studies on AI for dental implant identification (brand/type) via radiographs. Meta‐analysis found overall identification accuracy = 92.56% (90.49%–94.63% range). 11 studies had low risk of bias and 5 had high risk (QUADAS‐2). Results showed AI (CNN models) can accurately detect and classify implants on panoramic and periapical x‐rays. Low variability between studies' outcomes.	*AI is highly effective for identifying and categorizing implant systems on radiographs*. The authors conclude that AI‐based identification is sufficiently accurate (∼92%–93%) to be clinically useful for assisting in implant recognition. They recommend additional well‐controlled studies to determine which implant systems are most amenable to AI identification and to expand AI training on less‐common implant types. Future research should also address biases and ensure AI models generalize across different imaging conditions.
6. Alqutaibi et al.^18^	Included 12 studies on AI in implant treatment planning (edentulous site detection & bone assessment). Meta‐analysis reported pooled accuracy for detecting edentulous areas on imaging: 96% for mandible (95% CI: 94%–98%) and 83% for maxilla (CI: 82%–84%). Risk‐of‐bias assessment: 8 studies low risk, 2 some concerns, 2 high risk. AI models also measured bone dimensions and identified vital structures on CBCT.	*AI models can assist implant planning by accurately identifying available bone and edentulous spans*, with particularly excellent performance in the mandible. The authors highlight that AI could streamline digital treatment planning (e.g., automatically marking implant sites). However, the lower accuracy in maxillary sites suggests that AI may need enhanced training or imaging inputs for complex anatomy. They call for more research to improve generalizability and to integrate AI planning tools into clinical software, after further validation.
7. Alqutaibi et al.^20^	Covered 13 studies on AI for predicting implant prognosis (implant stability, failure, peri‐implantitis) from radiographic data. Reported performance: accuracy up to 99.8% in best cases, but sensitivity ranged 67%–95% and specificity 78%–100% across studies. AI significantly reduced analysis time versus manual for evaluating risk factors on images. Overall results varied due to different endpoints (bone loss, implant mobility, etc.) and AI methods.	*AI algorithms show promising capability in enhancing implant prognosis predictions and enabling earlier interventions*. The authors conclude that while some AI models achieved near‐perfect accuracy in detecting certain complications, the inconsistency among studies indicates that findings are not yet generalizable. They recommend standardizing outcome definitions (e.g., what constitutes “failure”) and improving model training with larger longitudinal datasets. Further research is needed before such AI predictions can be relied upon in routine follow‐up care.
8. Bonfanti‐Gris et al.^15^	Included 29 studies on AI in implant radiograph analysis: specifically, implant classification (identify implant type) and peri‐implant pathology detection. QUADAS‐2 assessment showed 10 studies high bias, 15 unclear, only 4 low bias. Found implant classification accuracy 67%–99% among studies (most above 85%). For peri‐implantitis detection, AI accuracy was generally ≥ 78.6%. Recommended exploring federated learning and advanced techniques to improve generalizability.	*AI (especially CNNs) performs at a high level in classifying implants and detecting peri‐implant disease on 2D radiographs*, but there are “several limitations” to address before clinical deployment. The authors emphasize the need for improving model robustness and training diversity—many included studies were single‐center with limited image variety. They suggest federated learning to gather broader data without privacy violations, as well as addressing class imbalance (some implant brands underrepresented). They conclude that while AI could aid clinicians in recognizing unknown implants and early peri‐implant bone loss, further development is required for consistent clinical reliability.
9. Macrì et al.^19^	Included 14 studies on AI for implant planning and anatomic structure identification (journal: Bioengineering). Key outcomes showed AI segmentations of the mandibular canal on panoramic/CBCT are highly accurate (one study: AI vs. manual overlap ∼85%); Maxillary sinus segmentation by CNN achieved high precision and consistency, enabling 3D models for virtual planning; AI‐based segmentation of alveolar bone and intraoral scans was 116× faster than manual (with only minimal accuracy trade‐off). Also, AI accurately segmented edentulous ridges (≥ 90% accuracy) for implant sites. No meta‐analysis due to heterogeneity. Quality of studies varied; authors noted lack of standard protocols.	*AI can greatly enhance implant treatment planning* by automatically and rapidly mapping patient anatomy (neural canals, sinus, bone topography) with accuracy comparable to clinicians. The authors conclude that these tools can reduce clinicians' workload and improve safety (e.g., avoiding nerve injury). However, they stress the need for high‐quality training data and standardized evaluation. Many studies used different metrics and lacked external validation, making direct comparison difficult. New studies should focus on clinical integration of these AI tools and on evaluating outcomes when clinicians use AI assistance versus not.
10. Dashti et al. ^13^	Systematic review and meta‐analysis on DL versus conventional algorithms for implant identification (13 studies, 3 in meta‐analysis). All included studies were rated low risk of bias. Meta‐analysis results: CNN models pooled accuracy ∼95.6%, sensitivity ∼94.5%, specificity ∼97.9%. Best‐performing CNN (Multitask ResNet152) had 99.08% accuracy. Conventional (non‐deep) models generally underperformed DL.	*State‐of‐the‐art DL models are extremely accurate in identifying dental implant types*, substantially outperforming older ML approaches. The authors suggest that these CNN models are approaching a ceiling of performance in controlled settings. They advocate for integrating such models into clinical workflows (e.g., software that assists radiographic diagnostics) given their proven accuracy. Additionally, they recommend expanding these models to recognize more implant types and testing them prospectively in clinics. The near‐perfect sensitivity/specificity achieved for certain brands implies AI could prevent identification errors that might occur manually.
11. Ibraheem et al.^16^	Systematic review (Diagnostics journal) on AI accuracy in implant fixture identification. Risk of bias showed 14 studies had some concerns (unclear or high in ≥1 domain), 7 were low‐risk. Out of 21 studies, most reported accuracy >90%, with a range from 67% (lowest) to 98.5% (highest). Many studies also reported high precision in classification tasks. Overall, evidence shows consistently high Performance of AI in identifying implant features on radiographs.	*AI tools achieve high accuracy in identifying and classifying dental implants from 2D radiographs*, validating their value as diagnostic aids. The review concludes that implementing these AI systems could improve clinical diagnostics by providing quick, reliable identification of implant types (useful, e.g., when treating a new patient with existing implants of unknown origin). However, they caution that many studies had methodological biases and were retrospective; thus, real‐world testing is needed. They also encourage researchers to address why certain cases (the ∼10% where AI misclassifies) fail, to further improve model training and performance.

**TABLE 4:** Selected top‐performing AI models and outcomes by clinical task in prosthodontics and implant dentistry.

Clinical domain	Specific AI task	Main AI models	Performance reported	Data source	Task‐level CCA	Clinical readiness
**General prosthodontic tasks**						
Diagnostics relevant to prosthodontic planning	Caries detection on periapical radiographs	Deep CNN	Accuracy for caries detection: premolars 89%, molars 88%, combined 82%, suggesting strong diagnostic support for evaluating abutment teeth.	Bernauer et al.^14^	CCA = N/A—only one data‐providing SR	Moderate
Diagnostics relevant to prosthodontic planning	Diagnosis/prediction of periodontally compromised teeth	CNN	Diagnostic accuracy around 81% (premolars) and 76.7% (molars) for identifying periodontally compromised teeth.	Bernauer et al.^14^	CCA = N/A—only one data‐providing SR	Moderate
**Tooth‑supported prosthodontic tasks**						
Fixed prosthodontics	Tooth shade selection	Back‐propagation neural network (BPNN)	AI shade‐matching system outperformed conventional visual methods in Δ*E* color difference	Revilla‐León et al.^6^ Bernauer et al.^14^	CCA = 1.00 (100%). Both reviews include the same single primary study	Moderate
Fixed prosthodontics	Automated restoration/tooth anatomy design	Knowledge‐based systems, statistical models, or computer vision methods	Among the better‐performing AI models, deviations between reconstructed and reference tooth surfaces were typically around 0.1–0.4 mm, with principal‐component models capturing ≈80% of the natural occlusal morphological variation.	Revilla‐León et al.^6^	CCA = N/A—only one data‐providing SR	Moderate
Fixed prosthodontics	Margin line/finishing‐line detection on prepared teeth	CNN‐based edge‐detection	CNN model located the preparation margin without manual interaction, with average accuracy 90.6%–97.4% across samples.	Revilla‐León et al.^6^	CCA = N/A—only one data‐providing SR	Moderate
Fixed prosthodontics	Debonding prediction for CAD/CAM composite crowns	CNN	98.5% accuracy for predicting debonding probability of CAD/CAM composite crowns in test images.	Bernauer et al.^14^	CCA = N/A—only one data‐providing SR	Emerging
Removable prosthodontics	RPD design—clinical decision support and automated CAD frameworks	Expert systems and knowledge‐based system	AI tools generally generated RPD designs judged clinically feasible; in one contemporary knowledge‐based CAD system, RPD designs were rated correct in 100% of mandibular and 75% of maxillary cases.	Revilla‐León et al.^6^	CCA = N/A—only one data‐providing SR	Emerging
**Implant prosthodontics and implant dentistry tasks**						
Fixed implant prosthodontics	Margin tracing and adaptation for implant‐supported monolithic zirconia crowns	Intrinsic AI features of commercial CAD software	The intrinsic AI margin‐tracing resulted in very accurate marginal adaptation in 96.2% of restorations.	Bernauer et al.^14^	CCA = N/A—only one data‐providing SR	Moderate
Implant identification	Implant type/brand/system identification and classification on 2D radiographs	Deep CNNs, transfer‐learning CNNs, classical ML algorithms	Across reviews, accuracy for implant system classification generally ranges 67%–99%; Pooled meta‐analysis found mean accuracy ≈95.6%, sensitivity ≈94.6%, specificity ≈97.9%.	Dashtiet al.^13^ Revilla‐León et al.^8^ Bernauer et al.^14^ Bonfanti‐Gris et al.^15^ Ibraheem et al. ^16^	CCA = N/A—Task‐level CCA cannot be reliably calculated without risking misclassification, because the SRs use different inclusion windows, language restrictions, and task stratifications	High
Peri‐implant health	Peri‐implantitis/peri‐implant bone‐loss detection on 2D radiographs	CNN	AI CNN models achieved accuracy ≥ 78.6% for peri‐implant pathology detection on 2D radiographs, and several individual studies reported diagnostic accuracies in the high 80%–90% range. However, reported metrics vary widely between studies due to differences in datasets, radiographic modality, and lesion definitions.	Bonfanti‐Gris et al.^15^	CCA = N/A—only one data‐providing SR	Emerging
Prognosis from imaging	Implant prognosis prediction from radiographic features	CNN‐based deep‐learning models and other ML algorithms	AI models showed promising ability to detect or predict marginal bone loss and peri‐implantitis from intra‐oral radiographs, achieving accuracies in the 80%–90% range and, where reported, AUCs ≥ 0.80; however, metrics, thresholds, and endpoints differ substantially across studies.	Bonfanti‐Gris et al.^15^ Alqutaibi et al.^20^	CCA = N/A—partially overlapping bone‐loss/peri‐implantitis studies are classified differently	Emerging
Prognosis from non‐radiographic factors	Implant success/failure prediction from risk factors and ontology‐based criteria	Various ML classifiers	AI models predicted implant success or complications with accuracies of roughly 62.4%– 80.5%, depending on the dataset and input features	Revilla‐León et al.^8^	CCA = N/A—only one data‐providing SR	Emerging
Implant planning on CBCT	Edentulous area segmentation and bone height/width measurement for implant site selection	U‐Net and 3D U‐Net variants, and CNN‐based models	AI models achieved pooled accuracies of 96% for detecting mandibular edentulous areas and 83% for maxillary edentulous areas on CBCT; bone‐dimension measurements were generally comparable to manual readings, with some site‐specific discrepancies.	Alqutaibi et al.^18^ Macrì et al.^19^	CCA = N/A—CCA cannot be reliably calculated because the two SRs categorized studies and performance metrics differently	High
Implant planning on CBCT	Anatomical landmark identification (inferior alveolar nerve, or ridge deficiencies)	3D U‐Net and other CNN‐based networks	Reported mandibular canal localization performance ranged from ∼77% accuracy to ∼99% precision when canal and alveolar bone were detected together, generally with clinically acceptable localization errors; ridge‐deficiency and related defect detection also showed promising but heterogeneously reported performance.	Macrìet al.^19^ Alqutaibi et al.^20^	CCA = N/A—task labels and overlapping landmark/ridge‐deficiency studies are categorized differently across SRs, so we can't reliably match them at task level.	High

*Note*: Performance metrics represent the best‐reported outcomes from each review; values may vary across individual primary studies. The final column indicates the clinical readiness of AI for each task, based on task‐level performance and evidence maturity.

Abbreviations: BPNN, backpropagation neural network; CAD‐CAM, computer‐aided design/computer‐aided manufacturing; CBCT, cone‐beam computed tomography; CCA, corrected covered area; CNN, convolutional neural network; ML, machine learning; RPD, removable partial denture; U‐Net, U‐Net convolutional neural network architecture.

To provide a clinically interpretable summary, an ordinal three‐level scale for the clinical readiness of AI (high, moderate, emerging) was constructed to reflect the current readiness of AI applications to support routine clinical care. For each clinical task in Table 4,^6^, ^8^, ^13^, ^14^, ^15^, ^16^, ^18^, ^19^, ^20^ the best‐performing metric reported in the source systematic review(s) (such as accuracy, area under the curve or AUC, sensitivity, specificity, or segmentation overlap) was extracted and classified as high (accuracy or AUC ≥ 0.90, sensitivity and specificity ≥ 0.90, or overlap ≥ 0.85), moderate (0.70–0.89, or overlap 0.70–0.84), or low (< 0.70, or overlap < 0.70); for shade‐matching and design tasks, performance was considered high when reported as clinically acceptable and superior to conventional methods. The maturity of the evidence base (number and consistency of primary studies, presence of meta‐analysis, risk‐of‐bias summaries, and use of real clinical data) was then judged qualitatively. High clinical readiness of AI was assigned when high performance was supported by a consistent evidence base (supported by multiple studies and at least one high‐quality systematic review, including meta‐analysis), moderate readiness when performance was high or moderate but evidence remained limited or heterogeneous, and emerging readiness when performance was low, highly variable, or clearly experimental. These ratings are also reported in Table 4.^6^, ^8^, ^13^, ^14^, ^15^, ^16^, ^18^, ^19^, ^20^

## RESULTS

### Search results

**FIGURE 1:**
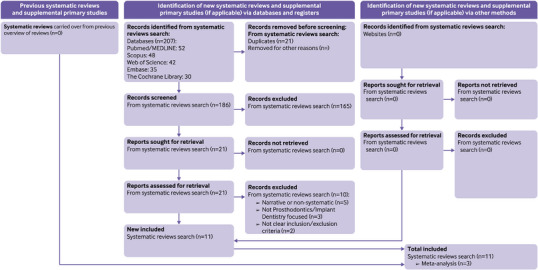
Flow diagram illustrating the literature selection process, adapted from PRISMA 2020 as recommended in the PRIOR reporting guideline. The review was reported according to the PRIOR 2022 guideline and registered in PROSPERO (CRD420251067048).

The comprehensive database search across PubMed, Scopus, Web of Science, Embase, and the Cochrane Database of Systematic Reviews initially retrieved 207 records. After duplicate removal using Mendeley software, 186 unique records underwent title and abstract screening. From these, 165 records were excluded based on irrelevance to the inclusion criteria, primarily due to a lack of systematic methodology, a focus on unrelated dental specialties, or inappropriate AI applications. Full texts of the remaining 21 articles were assessed in detail; ultimately, 11 systematic reviews satisfied all predefined eligibility criteria and were included for analysis. Ten articles were excluded at the full‐text screening stage due to non‐systematic methodologies, inappropriate applications of AI, or lack of relevance to prosthodontics or implant dentistry. Detailed citations and specific reasons for exclusion are provided in Table S1.^21^, ^22^, ^23^, ^24^, ^25^, ^26^, ^27^, ^28^, ^29^, ^30^ Figure 1 presents the detailed PRISMA flow diagram summarizing this selection process. Of the included systematic reviews, one was published in 2021, three in 2023, three in 2024, and four in 2025. Across the 11 included systematic reviews, we identified 281 primary‑study citations (counting each appearance of a primary study in any review). These corresponded to 261 unique primary studies (*r*) across the review corpus, yielding a CCA of 0.77%, indicating slight overlap and minimal risk of duplication bias affecting the overall results of this umbrella review. For comparison, the “Included studies (*n*)” column in Table 2 lists the number of studies included in the main analyses of each review (total = 193), which is a subset of the 281 citations used to calculate CCA. In addition, task‐level CCA was derived where feasible for individual clinical AI tasks and is reported in Table 4 to indicate the extent of primary‐study overlap between reviews for each synthesized outcome.

### Overview of included reviews

The 11 identified systematic reviews examine AI applications in prosthodontics and implant dentistry (Table 2). Collectively, these reviews cover a broad spectrum of topics. Three reviews (Table 2) focused on general prosthodontics (tooth‐supported prostheses and overall prosthodontic workflows), while the remaining eight (Table 2) concentrated on implant dentistry (AI in implant planning, identification, or prognosis). The number of primary studies included in each review ranged from as few as 7 up to 36, reflecting variability in how extensively each sub‐topic has been investigated to date. Notably, the earlier review by Bernauer et al. found only seven qualifying studies on AI in prosthodontics,^14^ whereas a later review included dozens of studies as the field rapidly expanded.^15^

Most reviews employed descriptive synthesis (73%). Meta‐analyses were conducted in three cases: Alqutaibi et al. pooled accuracy data for implant system identification,^9^ Alqutaibi et al. quantitatively synthesized diagnostic accuracy for edentulous site detection^18^ and bone measurements, and Dashti et al. quantitatively synthesized the diagnostic performance of DL models (CNNs) compared to other algorithms for implant identification.^13^ The other reviews provided narrative summaries or grouped analyses due to heterogeneity of methods and outcomes. All reviews conducted some form of quality/risk‐of‐bias assessment for their included studies (such as using QUADAS‐2 for diagnostic accuracy studies^9^, ^15^ or the JBI checklist for non‐randomized studies^6^). It was commonly noted that the quality of primary studies was mixed; many had high or unclear risk of bias (for instance, Bonfanti‐Gris et al. reported only 4 of 29 studies as low risk,^15^ and Ibraheem et al. found 14 of 21 with some risk of bias ^16^). Despite these limitations, all reviews pointed to consistent technical performance of AI systems in various prosthodontic tasks, albeit with cautions about the need for further validation.

For clarity, the findings were organized into two overarching domains: (1) AI in general prosthodontics (including tooth‐supported prostheses and broader prosthodontic workflows) and (2) AI in implant dentistry. Within each domain, the evidence was grouped by specific clinical application. Where available, key quantitative performance metrics (such as accuracy, sensitivity, specificity, or AUC) are reported together with the corresponding source review. Table 3 synthesizes the major outcomes and conclusions from each review, covering both prosthodontic and implant‐focused applications. It highlights the consistency of evidence (such as all reviews on implant identification agree on high accuracy) and also notes variability where present (such as prognostic accuracy). Reported statistics from the original systematic reviews (such as accuracy values, sensitivity, specificity, etc.) are cited throughout to illustrate and support the synthesis of findings. Table 4 highlights selected top‐performing AI models for each clinical task, along with their reported performance and data sources. Based on the predefined performance bands (high, moderate, low) and a qualitative judgement of evidence maturity (number and consistency of contributing studies, presence of meta‐analysis, and risk‐of‐bias summaries), each task was assigned one of three levels of clinical readiness of AI (high, moderate, emerging). Overall, the findings indicate that AI is a valuable adjunct in prosthodontics and implant dentistry, particularly for radiographic diagnostics and CBCT‐based treatment planning, and shows early promise in predictive modelling and prosthesis design.

### AI applications in general prosthodontics (tooth‐supported prostheses and overall prosthodontic workflows)

Three systematic reviews evaluated AI within traditional prosthodontics, involving prostheses supported by natural teeth or mucosa and excluding surgical implant placement.^6^, ^14^, ^17^ These reviews addressed diverse applications, from CAD‐CAM‐driven workflows to diagnostic support and educational tools. Revilla‐León et al. provided the most structured synthesis, categorizing six primary tasks where AI has been applied in tooth‐supported prosthodontics.^6^

In the area of tooth shade selection, AI‐driven matching systems have been shown to reduce subjectivity by offering consistent, reproducible results. One study identified by Revilla‐León et al. demonstrated that an AI model outperformed visual shade selection methods, achieving more accurate matches to target dentition.^6^ Regarding automated restoration design, fourteen studies, in Revilla‐León et al., explored the use of CNNs and generative models to create digital prosthetic designs such as crowns, inlays, and partial fixed dental prostheses (PFDP). These models reliably generated anatomically appropriate restorations and demonstrated fabrication success comparable to human‐designed equivalents, particularly in simulation or in vitro settings.^6^ Preparation margin identification has also benefited from AI integration. One model was able to detect finish lines on tooth preparations with accuracy ranging between 90.6% and 97.4%, reducing the need for manual delineation and thereby improving workflow efficiency. For manufacturing process optimization, AI was used to refine casting protocols for metal frameworks, minimizing defects and porosity while also reducing fabrication time. These improvements could enhance the quality of frameworks for removable prostheses.^6^

In more specialized domains, AI has been explored for facial prosthesis outcome prediction, particularly in maxillofacial prosthodontics. One reviewed study used AI to predict facial soft tissue changes in patients receiving removable prostheses. Although quantitative metrics were not specified, the system produced clinically relevant outcomes that could support aesthetic planning.^6^ The most extensive application was found in removable partial dental prosthesis (RPD) design and educational tools. Seventeen studies described AI systems that supported RPD framework design by analyzing edentulous areas and abutment conditions while also serving as interactive teaching platforms. These tools encoded expert knowledge from clinical texts and cases, facilitating both treatment planning and dental education.^6^

Overall, these reviews indicate that AI models have demonstrated diagnostic and predictive performance comparable to experts in several core prosthodontic tasks. For example, matching tooth shades and identifying preparation margins were both performed with high reliability.^6^, ^17^ However, the evidence base remains relatively small. Bernauer et al. included only seven studies in their 2021 review, limited to those with five patients or more, underscoring that clinical AI use was still in early stages at that time.^14^ The rapid pace of digital innovation means that AI models developed on earlier‐generation imaging and workflow data may not remain optimal indefinitely, highlighting the need for periodic validation and potential updating or replacement. More recently, Maktabi et al. observed broader adoption of AI in prosthodontics, noting its role in improving treatment efficiency and reducing error rates, especially in implant‐related applications, likely due to the abundance of digital imaging data available in that context.^17^

### AI applications in implant dentistry

Eight systematic reviews focused on implant dentistry, covering AI applications in implant system identification, treatment planning, outcome prediction, disease detection, and implant design. The findings across these domains were largely complementary.

Implant system identification and classification was consistently reported as a mature application of AI. Reviews by Alqutaibi,^9^ Dashti,^13^ Ibraheem,^16^ and Bonfanti‐Gris^15^ demonstrated that deep CNNs accurately identified implant types from radiographs. Alqutaibi et al. reported pooled accuracy of 92.56%,^9^ while Dashti et al. found 95.6% with sensitivity and specificity approaching 95% and 98%,^13^ respectively. Top‐performing models like ResNet‐152 and Neuro‐T v2.0.1 achieved up to 99.08% and 100% sensitivity. Ibraheem found most studies exceeded 90% accuracy, with a range of 67%–98.5%.^16^ Bonfanti‐Gris noted 67%–99% accuracy using 2D radiographs.^15^ Collectively, these reviews support AI as a reliable tool for implant identification, matching or exceeding expert performance, though most studies used idealized datasets and generalizability requires further validation.

Implant treatment planning and anatomy evaluation is another key area. Alqutaibi et al. reported that AI detected edentulous areas on radiographs with 96% accuracy in the mandible and 83% in the maxilla.^18^ Macrì et al. highlighted CNN models for automatic segmentation of vital structures, such as the mandibular canal and maxillary sinus, using 3D U‐Net models.^19^ These tools delivered high accuracy and dramatic time savings. In one study, AI segmented bone crests 116 times faster than manual efforts. While direct implant positioning was less explored, these tools significantly enhance preoperative planning precision.^19^

Implant success and prognosis prediction were reviewed by Alqutaibi et al. and Revilla‐León et al.^8^, ^20^ AI algorithms predicted outcomes like implant failure and peri‐implantitis from radiographs with accuracy up to 99.8%, although sensitivity and specificity varied widely (67%–95% and 78%–100%). Models based on patient and biomechanical data yielded lower accuracy (62.4%–80.5%), reflecting the complexity of multifactorial prediction tasks. Both reviews called for larger datasets and unified modelling standards to improve predictive robustness.^8^, ^20^

Peri‐implant disease detection was addressed by Bonfanti‐Gris et al.,^15^ who reported AI models achieved at least 78.6% accuracy in detecting peri‐implant radiolucencies on 2D images. While accuracy varied, the potential for early detection and automated monitoring was clear. The authors advocated federated learning approaches to improve model generalizability across clinical environments. AI for implant design optimization was reviewed by Revilla‐León et al.,^8^ who examined studies combining AI with finite element analysis. These systems optimized implant geometries, reducing stress at the bone–implant interface by up to 36.6%. Though still in experimental stages, these findings suggest that AI could play a transformative role in future implant product development.

### Cross‐cutting findings

Across reviews, AI achieved high accuracy for recognition and segmentation tasks in well‐defined settings. These include implant type identification on radiographs, anatomic segmentation for planning, and specific prosthodontic tasks such as preparation margin detection and objective shade matching.^6^, ^8^, ^9^, ^13^, ^15^, ^18^, ^19^ Performance was more modest or variable for multivariate prognosis based on patient or biomechanical factors and for maxillary edentulous site detection, reflecting endpoint heterogeneity and data limitations.^8^, ^18^, ^20^ These performance patterns were contextualized by task‐level evidence summarized in Table 4. For most clinical tasks, only a single data‐providing systematic review was available, or the contributing reviews categorized overlapping primary studies too differently to allow reliable matching; in these situations, task‐level CCA is reported as not applicable (N/A) together with a brief justification. The main exception is tooth shade selection, for which two reviews relied on the same single primary study (task‐level CCA = 1.00). Overall, the reported CCA values indicate that, at the level of individual tasks, the evidence summarized in different reviews is based on distinct or only minimally overlapping sets of primary studies, emphasizing that task‐specific performance estimates should be interpreted in light of their underlying evidence base rather than as pooled effects across reviews.

However, all reviews caution that many primary studies were conducted in ideal settings, such as in vitro or single‐center designs. This limits generalizability, and significant methodological heterogeneity further complicates interpretation. In prognostic models, outcome definitions varied, some predicting implant survival, others focusing on marginal bone loss thresholds, making direct comparisons difficult. This heterogeneity was reflected in risk‐of‐bias assessments, with numerous studies flagged for unclear or high risk due to issues like limited validation or lack of blinding.^15^, ^16^ Notably, some primary studies appeared in multiple reviews, especially in implant identification, yet consistent conclusions emerged. For example, implant ID studies featured across three reviews all reported high accuracy, reinforcing the robustness of these findings.^14^ Differences in reported accuracy between reviews (such as 92.56% in Alqutaibi et al.^9^ vs. 95.6% in Dashti et al.^13^) likely reflect differences in included studies or model improvements over time, rather than disagreement. Another recurring theme is the urgent need for standardization in AI evaluation. Macrì et al. highlighted inconsistent reporting,^19^ which hampers cross‐study comparisons. Likewise, Schwendicke et al., cited in Maktabi, noted variability in outcome measures and metrics.^17^ Many reviews advocate for adopting common performance indicators (such as F1‐score, AUC, or diagnostic accuracy) and establishing shared benchmark datasets to ensure reproducibility and comparability. Without such standardization, the evidence base remains fragmented and difficult to synthesize effectively.

### Quality of the systematic reviews

Based on the AMSTAR‐2 tool, of the 11 systematic reviews assessed, 36% (4/11) were rated as high quality, 9% (1/11) as low quality, and 55% (6/11) as critically low quality, primarily due to repeated failures in critical domains (Table 2 and Table S2). Notably, 100% of the reviews failed to report the sources of funding for the included studies (Item 10). Moreover, 91% did not provide a list of excluded studies with justifications (Item 7), and 36% failed to perform or report study selection in duplicate (Item 5). The absence of a predefined protocol (Item 2) was also a recurrent issue, with only 4 out of 11 reviews (36%) providing PROSPERO registration or equivalents.

## DISCUSSION

### Overview and consistency of findings

This umbrella review combined evidence from 11 systematic reviews, encompassing 261 primary studies, to provide a big‐picture assessment of AI in prosthodontics and implant dentistry. The results offer both encouraging validation of AI capabilities and a tempered understanding of current limitations and research needs.

One striking aspect is the consistency of positive findings across independent reviews. Multiple reviews, often with overlapping but not identical sets of studies, arrived at the conclusion that AI systems (particularly DL models) perform extraordinarily well in well‐defined prosthodontic tasks. For example, four separate reviews (Alqutaibi et al,^9^ Bonfanti‐Gris et al.,^15^ Dashti et al,^13^ Ibraheem et al.^16^ in Table 2) looked at AI identifying implant brands via radiographs and all reported accuracies in the 90%–99% range.^9^, ^13^, ^15^, ^16^ This convergence greatly increases confidence that this is a real, robust capability of AI that has been reproduced by different groups. Another example is AI for anatomic segmentation in implant planning: both an oral radiology‐focused review^19^ and a more general implant planning review^18^ emphasize that CNNs can reliably map out critical anatomy (such as nerve canals, maxillary sinuses, and bone topography) and measure bone with high accuracy and speed. The agreement between these sources, despite minor differences in exact metrics, suggests the technology is indeed effective and not dataset‐specific. Where findings differ, it often reflects differences in scope rather than contradiction. For instance, Revilla‐León's implant review found moderate accuracy (60%–80%) for AI predicting implant success using patient factors,^8^ whereas Alqutaibi's prognosis review found some models hitting 95% or higher accuracy using radiographic analysis.^20^ At first glance, these could be seen as conflicting, but in reality they address different modelling approaches: one predicts long‐term outcomes from clinical factors, the other diagnoses current implant status from images. Each is valid in its context; taken together they indicate that image‐based detection of existing problems is more mature than holistic prediction of future implant fate. Thus, rather than conflict, these findings map the landscape of what AI does well now (diagnosis/recognition tasks) versus what is still challenging (multi‐factor prognostication). While this umbrella review synthesized findings primarily by clinical application, the type of AI models used, such as CNNs, ANNs, and ensemble methods, was documented for each task and summarized in Table 4. This provides a foundation for future reviews to perform deeper comparative analysis of AI architecture‐specific performance. A thematic synthesis organized by model type may help identify which AI approaches consistently outperform others across varied clinical contexts.

### Limitations of included evidence

Despite the encouraging performance metrics, the underlying evidence has important limitations frequently noted by the original review authors. A recurring theme is that many primary studies were retrospective, single‐center, or had small sample sizes. As reported, a large fraction of studies had a high or unclear risk of bias.^15^, ^16^ Common issues included using retrospective datasets without prospective validation, lack of blinding (some studies might have had the algorithm's developers assessing outcomes), and sometimes incomplete reporting of data handling. These factors mean that the reported accuracies, while high (near 100% sensitivity or 97% specificity),^15^, ^16^ may reflect idealized research conditions. The reviews uniformly call for caution, that we should not assume these numbers will directly translate to every clinic's reality. For example, the best‐performing implant identification CNN might have been trained on high‐resolution, controlled radiographs predominantly featuring a few implant brands; in practice, radiographs vary in contrast, angulation, presence of restorations, and so forth, and a clinic may see dozens of different implant brands (including ones not in the training set). The algorithm's real‐world accuracy could thus be lower. The need for external validation of AI models is highlighted: none of the systematic reviews found much evidence of AI models validated on entirely independent cohorts (an aspect often lacking in early AI studies).

Another limitation is scarcity of longitudinal and clinical outcome data. Current research emphasizes accuracy measures and laboratory metrics, yet few investigations assess whether AI use actually improves patient outcomes or procedural efficiency in practice. For instance, although AI can design an RPD framework, no study has shown that dentists relying on AI‐generated designs produce better‐fitting dentures or save chairside adjustment time compared with traditional methods. Addressing these gaps is vital for progressing from technological demonstration to clinical implementation. The methodological quality of the included systematic reviews was mixed: although a minority were rated as high quality, most were low or critically low according to AMSTAR‐2, primarily due to missing protocols, incomplete reporting of excluded studies, and limited funding transparency (Table 2). This limits the certainty of the umbrella‐level conclusions and reinforces the need for more rigorously conducted future reviews.

It is worth noting that two of the most crucial topics, implant identification and implant planning, were each addressed by multiple high‐quality reviews, which reduces the chance that any single review's bias or omissions affected our conclusions. One review by Maktabi et al. had some methodological shortcomings (unclear selection criteria, likely omission of relevant observational studies, and reliance on a small subset of evidence),^17^ but fortunately its conclusions do not contradict those of better‐quality reviews. In fact, Maktabi's conclusions were generally aligned (stating that AI is beneficial in all areas of prosthodontics and especially implant dentistry), albeit without the depth of analysis others provided. This points to a redundancy in the literature, multiple systematic reviews on overlapping themes, which is both a strength (for verification) and a weakness (potential duplication of effort).

### Clinical and educational implications

For prosthodontists and implant surgeons, this umbrella review offers a cautiously optimistic message: AI tools are approaching readiness for clinical integration. Clinicians should prepare for workflows where an AI algorithm pre‐screens radiographs to mark peri‐implant bone loss or identify implant systems, potentially reducing workload and facilitating earlier diagnosis.^7^, ^15^ However, clinicians must remain critical and not over‐rely on AI without verification. Practically, AI could serve effectively as a quality‐assurance tool, similar to double‐reading in radiology, helping to confirm findings or flag discrepancies for closer inspection.

For tasks like prosthesis design, AI might initially serve in a supportive capacity. For example, CAD software could suggest crown morphology via AI, which dental technicians can then refine, accelerating the design phase. Clinicians and technicians will need to develop new skills combining dental anatomy expertise with digital design tools. Encouragingly, evidence indicates AI‐generated designs typically require only minor adjustments, yet human oversight remains crucial to ensure quality.^6^

There are also implications for training new dentists. As AI assumes routine tasks like landmark identification or simple restoration designs, dental education may shift towards supervising AI outputs and complex decision‐making skills that AI cannot replace. AI‐driven educational tools, as observed in RPD design training programs, could enhance students' learning outcomes.^6^ Educators should integrate these technologies while ensuring students grasp underlying principles to avoid over‐reliance.

### Research gaps and future directions

To address current evidence gaps, future research should prioritize several key areas. Prospective clinical trials are necessary to assess AI's real‐world impact on surgical outcomes, patient satisfaction, and clinical efficiency, comparing AI‐assisted methods with conventional approaches. The development of standardized benchmarks, such as shared datasets and competitive challenges for tasks like implant identification, would enable objective model comparison and foster improvements.

Multicenter and federated studies are also crucial. Training AI models across diverse populations and clinical settings, without compromising patient privacy, would improve generalizability. Federated learning methods can facilitate collaborative model training without data sharing, significantly enhancing cybersecurity.^15^ Ensuring diverse representation, including ethnic and cultural variations, is particularly important for applications involving facial prosthetic design.

Another underexplored but essential area is AI explainability. Models should clearly communicate their predictions and underlying rationale, enhancing clinician trust and informed decision‐making. Research should evaluate clinicians' interpretation and integration of AI outputs, identifying any necessary training enhancements. Lastly, systematic exploration of AI integration within digital dentistry workflows, including CAD‐CAM, intraoral scanning, and robotic surgical systems, will be critical. Evaluating whether AI outputs require minimal manual adjustments or can be directly utilized will determine the practical implementation success.

### Limitations of this umbrella review

Limitations of this umbrella review include a strict focus on prosthodontics and implant dentistry, potential publication bias favoring positive results, and the possibility of emerging evidence post‐search date (April 30, 2025). Funding sources for included studies were inconsistently reported, limiting assessment of funding‐related bias. Substantial methodological heterogeneity precluded meta‐analysis at the umbrella review level. While diagnostic and predictive AI applications warranted frameworks like QUADAS‐2 and AMSTAR‐2, GRADE assessment was less applicable. Lastly, umbrella reviews inherently involve interpreting prior interpretations; thus, direct accuracy metrics and clearly reported outcomes were prioritized to minimize distortion.

## CONCLUSION

In prosthodontics and implant dentistry, current AI systems generally show high technical and often clinically acceptable performance for specific recognition and segmentation tasks, particularly in radiographic implant identification and CBCT‐based planning, while evidence for prognostic and more complex decision‐making applications remains limited and heterogeneous. Because most data originate from systematic reviews with low or critically low methodological quality, these findings should be interpreted with caution. At present, AI tools are best viewed as adjunctive “intelligent assistants” that can support, but not replace, clinician judgment and conventional workflows.

## Supporting information



Supporting Information

## Data Availability

Data sharing not applicable to this article as no datasets were generated or analyzed during the current study.
