# Supplementary material for: Harnessing AI in prosthodontics and implant dentistry: An umbrella review of systematic evidence
Source: J Prosthodont. 2026 Jan 14;35(2):127–42. doi: 10.1111/jopr.70091 (PMC12906322; doi:10.1111/jopr.70091)
Supplement: Supplementary file 1 — Supporting Information [file JOPR-35-127-s001.docx]

Supplementary Table 1. List of excluded articles at the full-text screening stage

| **No.** | **Citation** | **Reason for Exclusion** |
| --- | --- | --- |
|  | Brahmi et al. Exploring the Role of Convolutional Neural Networks (CNN) in Dental Radiography Segmentation: A Comprehensive Systematic Literature Review ^21^ | Focuses on general dental radiography segmentation; lacks specific application to prosthodontics or implant dentistry. |
|  | Sarwar et al. AI Techniques for Cone Beam Computed Tomography in Dentistry: Trends and Practices ^22^ | Discusses AI in CBCT imaging broadly; does not specifically address prosthodontic or implant applications. |
|  | Villena et al. Generative Artificial Intelligence in Dentistry: Current Approaches and Future Challenges ^23^ | Explores generative AI in dentistry without a focus on prosthodontics or implant dentistry. |
|  | Wang et al. Artificial Intelligence to Assess Dental Findings from Panoramic Radiographs—A Multinational Study ^24^ | Concentrates on AI assessment of panoramic radiographs; lacks direct relevance to prosthodontic or implant procedures. |
|  | ElShamally HE. AI and Digital Dentistry in Prosthodontics: A Systematic Review ^25^ | While titled as a systematic review, the methodology lacks rigor, and the AI applications discussed are not directly pertinent to prosthodontics or implant dentistry. |
|  | Banerjee et al. Unveiling the Prospects and Challenges of Artificial Intelligence in Implant Dentistry: A Systematic Review^26^ | Although focused on implant dentistry, the review includes studies with diverse AI applications, some of which are not directly related to prosthodontics or implant dentistry. |
|  | Koul R et al. Artificial Intelligence in Prosthodontics: Current Applications and Future Avenues—A Narrative Review^27^ | Narrative review; does not meet systematic review criteria. |
|  | Kong et al. Application of Artificial Intelligence in Dental Crown Prosthesis: A Scoping Review^28^ | Scoping review; lacks systematic methodology and specific focus on prosthodontic or implant applications. |
|  | Benankatti et al. Artificial intelligence applications in dental implantology: A narrative review^29^ | Narrative review; does not meet systematic review criteria. |
|  | Farhadi Nia et al. Transforming dental diagnostics with artificial intelligence: advanced integration of ChatGPT and large language models for patient care^30^ | Discusses AI applications in general dental diagnostics; lacks specific focus on prosthodontics or implant dentistry. |

Supplementary Table 2. Results of the AMSTAR-2 critical appraisal of the included systematic reviews.

| **AMSTAR Item** | **Bernauer et al.**  **(2021) ^14^** | **Revilla-León et al.**  **(2023)*^6^*** | **Maktabi et al.**  **(2023) ^17^** | **Revilla-León et al.**  **(2023)*^9^*** | **Alqutaibi et al.**  **(2025) ^10^** | **Alqutaibi et al.**  **(2025) ^18^** | **Alqutaibi et al.**  **(2024) ^20^** | **Bonfanti-Gris et al.**  **(2025) ^15^** | **Macrì et al.**  **(2024) ^19^** | **Dashti et al.**  **(2025) ^8^** | **Ibraheem et al.**  **(2024) ^16^** |
| --- | --- | --- | --- | --- | --- | --- | --- | --- | --- | --- | --- |
| **TOTAL SCORE** | **CRITICALLY LOW** | **CRITICALLY LOW** | **LOW** | **CRITICALLY LOW** | **HIGH** | **HIGH** | **HIGH** | **HIGH** | **CRITICALLY LOW** | **CRITICALLY LOW** | **CRITICALLY LOW** |
| 1. Did the research questions and inclusion criteria for the review include the components of PICO? | Yes | Yes | No | Yes | Yes | Yes | Yes | Yes | Yes | Yes | Yes |
| 2. Did the report of the review contain an explicit statement that the review methods were established prior to the conduct of the review and did the report justify any significant deviations from the protocol? | No | No | No | No | Yes  (PROSPERO:  CRD42023479106) | Yes  (PROSPERO:  CRD42024590334 | Yes  (PROSPERO:  CRD42023482364 | Yes  (PROSPERO:  CRD42024575604 | No | No | Yes  (PROSPERO:  CRD42024500347 |
| 3. Did the review authors explain their selection of the study designs for inclusion in the review? | Yes | Yes | Yes | Yes | Yes | Yes | Yes | Yes | Yes | Yes | Yes |
| 4. Did the review authors use a comprehensive literature search strategy? | Yes | Yes | No | Yes | Yes | Yes | Yes | Yes | Yes | Yes | Yes |
| 5. Did the review authors perform study selection in duplicate? | NR | NR | NR | NR | Yes | Yes | Yes | Yes | Yes | Yes | Yes |
| 6. Did the review authors perform data extraction in duplicate? | Yes | Yes | NR | Yes | Yes | Yes | Yes | Yes | NR | Yes | Yes |
| 7. Did the review authors provide a list of excluded studies and justify the exclusions? | No list was provided, only reasons. | No list was provided, only reasons. | No | No list was provided, only reasons. | Yes | Yes | Yes | Yes | No list was provided, only reasons. | No list was provided, only reasons. | No list was provided, only reasons. |
| 8. Did the review authors describe the included studies in adequate detail? | Yes | Yes | No | Yes | No | No | No | Yes | Yes | Yes | Yes |
| 9. Did the review authors use a satisfactory technique for assessing the risk of bias (RoB)? | Yes | Yes | No | Yes | Yes | Yes | Yes | Yes | Yes | Yes | Yes |
| 10. Did the review authors report on the sources of funding for the studies included? | No | No | No | No | No | No | No | No | No | No | No |
| 11. If meta-analysis was performed, did the review authors use appropriate methods? | / | / | / | / | / | Yes | Yes | / | / | Yes | / |
| 12. If meta-analysis was performed, did the review authors assess the impact of RoB? | / | / | / | / | / | Yes | Yes | / | / | Yes | / |
| 13. Did the review authors account for RoB in individual studies when interpreting/discussing the results? | No | Yes | No | Yes | Yes | Yes | Yes | Yes | Yes | Yes | Yes |
| 14. Did the review authors provide a satisfactory explanation/discussion of heterogeneity? | Yes | Yes | No | Yes | Yes | Yes | Yes | Yes | Yes | Yes | Yes |
| 15. If quantitative synthesis was performed, did the review authors carry out an adequate investigation of publication bias? | / | / | / | / | / | Less than 5 studies were included in the meta-analysis. | Less than 5 studies were included in the meta-analysis. | / | / | Less than 5 studies were included in the meta-analysis. | / |
| 16. Did the review authors report any potential sources of conflict of interest? | Yes | Yes | Yes | Yes | Yes | Yes | Yes | Yes | Yes | Yes | Yes |
